# Deep learning-based classification of the mouse estrous cycle stages

**DOI:** 10.1038/s41598-020-68611-0

**Published:** 2020-07-16

**Authors:** Kyohei Sano, Shingo Matsuda, Suguru Tohyama, Daisuke Komura, Eiji Shimizu, Chihiro Sutoh

**Affiliations:** 1 Department of Cognitive Behavioral Physiology, Chiba University Graduate School of Medicine, 1-8-1 Inohana, Chiba, Chiba 260-8670 Japan; 2 Department of Pharmacotherapeutics, Showa Pharmaceutical University, 3-3165, Higashi-Tamagawagakuen, Machida, Tokyo 194-8543 Japan; 3 Department of Ultrastructural Research, National Institute of Neuroscience, National Center of Neurology and Psychiatry, 4-1-1 Ogawahigashi, Kodaira, Tokyo 187-8502 Japan; 4 Department of Preventive Medicine, Graduate School of Medicine, The University of Tokyo, 7-3-1 Hongo, Bunkyo-ku, Tokyo 113-0033 Japan

**Keywords:** Endocrine system and metabolic diseases, Diagnostic markers, Microscopy

## Abstract

There is a rapidly growing demand for female animals in preclinical animal, and thus it is necessary to determine animals' estrous cycle stages from vaginal smear cytology. However, the determination of estrous stages requires extensive training, takes a long time, and is costly; moreover, the results obtained by human examiners may not be consistent. Here, we report a machine learning model trained with 2,096 microscopic images that we named the "Stage Estimator of estrous Cycle of RodEnt using an Image-recognition Technique (SECREIT)." With the test dataset (736 images), SECREIT achieved area under the receiver-operating-characteristic curve of 0.962 or more for each estrous stage. A test using 100 images showed that SECREIT provided correct classification that was similar to that provided by two human examiners (SECREIT: 91%, Human 1: 91%, Human 2: 79%) in 11 s. The SECREIT can be a first step toward accelerating the research using female rodents.

## Introduction

Knowledge of the precise stages of the estrous cycle is very important for interpretations of female animals' data. Compared to men, women have a high lifetime incidence of several mental illnesses, including depression, post-traumatic stress disorders, generalized anxiety, and eating disorders^1^, but preclinical animal investigations for these illnesses have use mainly males in part because of the volatility of female animals' experimental data that cannot be separated from their estrous cycle. Several research groups have stated that it is difficult to apply the findings from males' preclinical results to women's medicine^2^. Indeed, the estrous cycle affects the expression of genes^3^, proteins^4,5^, electrophysiological properties^6,7^, behaviors^8,9^, and drug effects^10^. In 2015, the U.S. National Institutes of Health (NIH) announced that when conducting NIH-funded research, researchers should study both sexes^11^. The number of preclinical studies using female animals is thus gradually increasing.

**Figure 1:**
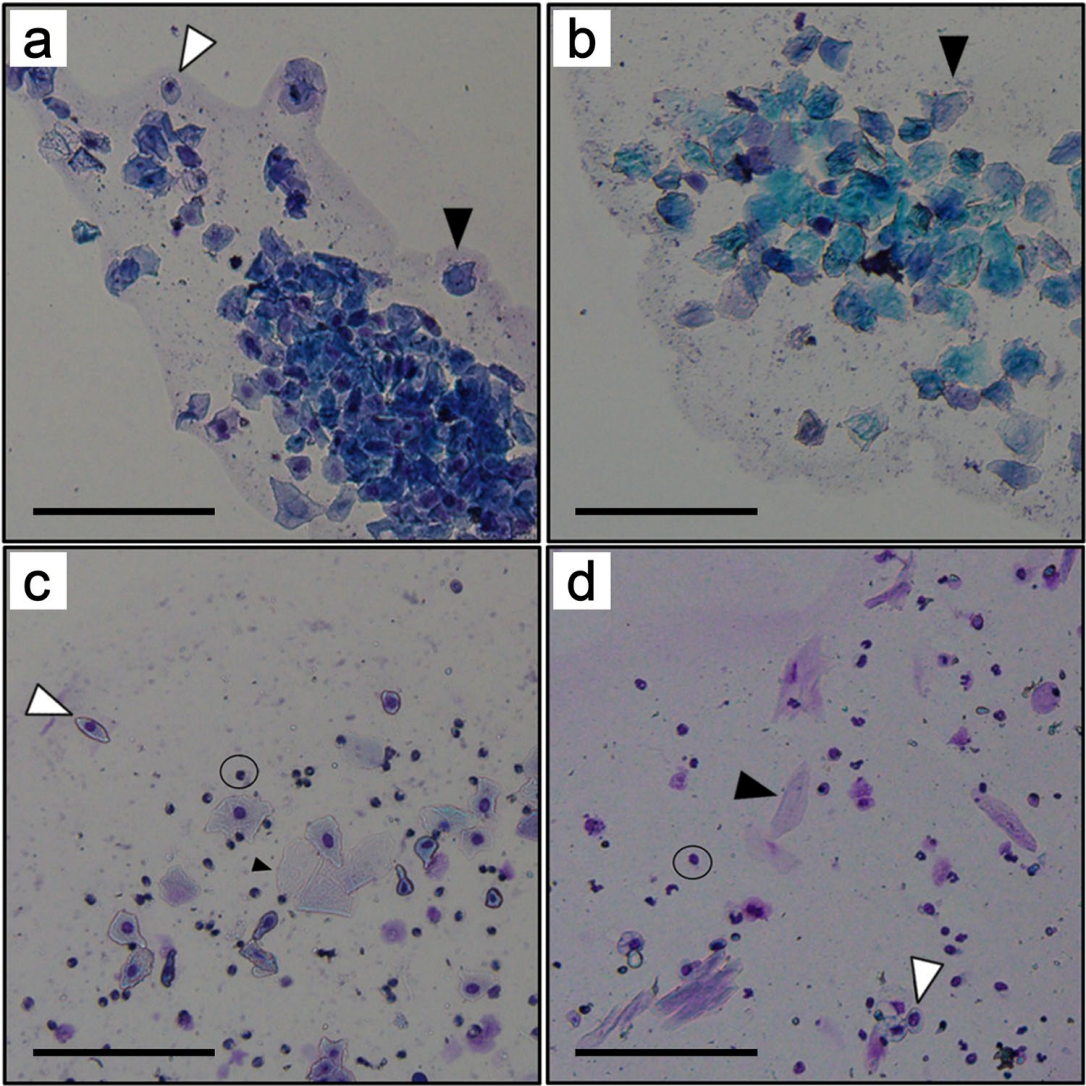
Vaginal cytology presenting each stage of the mouse estrous cycle. Three cell types are identified in vaginal smear images: leukocytes (circles), cornified epithelial cells (black arrowheads), and nucleated epithelial cells (white arrowheads). Stages of the estrous cycle include the (**a**) proestrus (P), (**b**) estrus (E), and (**c**) diestrus (D) stages. (**d**) An image of a vaginal sample from an ovariectomized female mouse. Scale bars represent 100 μm.

The estrous cycle in rodents is generally divided into three or four stages^12^, and the cycle is 4–5 days: Diestrus (D) → proestrus (P) → estrus (E) → (metestrus) → . In many studies of rodents, the estrous cycle stage of each animal has been determined by vaginal cytology. Each stage is decided based on the type, number, shape, size, and proportion of cells in a vaginal smear^12–17^ (Fig. 1a–c). Briefly, stage D was identified by the presence of leukocytes and nucleated cells with or without a few cornified cells. Stage P was identified by the presence of nucleated epithelial cells and cornified cells without leukocytes. Stage E was identified by the presence of nucleated epithelial cells without leukocytes or cornified cells. Vaginal cytology is also used to decide whether an ovariectomy in a rodent is successful^18,19^ (Fig. 1d).

However, determining the estrous stage of a rodent by using vaginal cytology evaluated by a human examiner has some problems: (1) a long training period is required in order to become skillful; (2) it takes a long time to determine the estrous stage from images, and doing so can be costly; and (3) the evaluations sometimes do not fully match among human examiners.

Computer-aided estrous stage classification is a potential solution to these problems. Machine learning algorithms powered by computational advances and large-scale datasets have provided dramatic progress, especially in visual tasks such as object recognition and visual classification. Such algorithms have also been applied to medical fields, and they have performed comparably or better than humans in some fields including the diagnosis of skin rashes^20^, and the evaluations of chest X-rays^21^ and histopathological images^22,23^. In the present study, we developed a classifier of estrous stage using machine learning and named it the "Stage Estimator of estrous Cycle of RodEnt using an Image-recognition Technique (SECREIT)". We applied deep learning algorithms^24^ to the automatic classification of the estrous stages (D, P, and E) in mice, and we observed that SECREIT performed comparably to skilled human examiners.

## Results

To evaluate SECREIT's performance, we adopted a hold-out validation. We calculated the sensitivity, specificity, receiver-operating-characteristic (ROC) curve, and AUC in a test dataset for each estrous stage. We tested two neural network architectures: VGG16 with 15 layers and CBR-LargeT with 6 layers, which was developed for medical image classification tasks. VGG16-based model achieved high AUCs (> 0.950) in all stages and consistently outperformed the CBR-LargeT model (Tables 1, 2, Supplementary Fig. S1). Thus, we adopted the VGG16-based model as the SECREIT model. We also observed that the SECREIT model showed higher sensitivity for the D and E stages than for the P stage, and the specificity values were constantly high for all three stages (Table 1).

**Table 1:** Model performance in 736 test images.

Model	D	P	E	Overall accuracy
**SECREIT (VGG16-based model)**				93.3%
Sensitivity	95.9%	74.6%	93.4%	
Specificity	94.0%	98.1%	96.2%	
AUC	0.982	0.962	0.979	
**CBR-LargeT**				84.9%
Sensitivity	84.6%	61.2%	94.5%	
Specificity	96.0%	93.9%	89.2%	
AUC	0.962	0.885	0.973

**Table 2:** Confusion matrix of estrous stage classification by SECREIT and CBR-LargeT model using 736 test images.

Ground truth	SECREIT (VGG16-based model)			CBR-LargeT			Total
	D	P	E	D	P	E	
D	466	2	18	411	34	41	486
P	14	50	3	7	41	19	67
E	1	11	171	3	7	173	183

**Figure 2:**
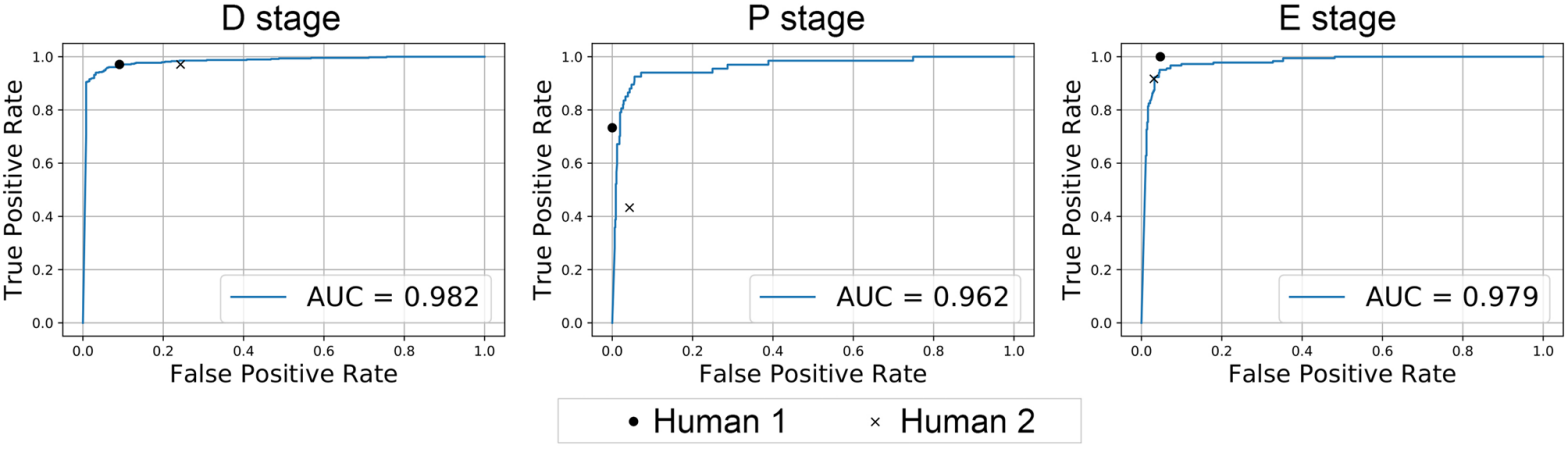
Comparison of the accuracy indices for SECREIT and two skilled human examiners. The ROC curves for the SECREIT and true positive rate and false positive rate by the two human examiners are illustrated. *AUC* area under the curve.

We next compared the performance of estrous stage classification among the SECREIT and two skilled examiners using the randomly sampled 100 images. SECREIT, Human 1, and Human 2 achieved 91%, 91%, and 79% overall accuracy, respectively (Tables 3, 4). The misclassification pattern of SECREIT was similar to that of Human 1, and seven of the nine misclassifications by SECREIT were the same misclassifications as those made by Human 1 or Human 2. As shown in Table 4, the sensitivity and specificity of SECREIT for the D and E stages were comparable to those of Human 1 and Human 2. The sensitivity of SECREIT for stage P was higher than those of Humans 1 and 2, and the specificity was comparable to those of Humans 1 and 2. The ROC curves also revealed that the performance of SECREIT was comparable to those of Humans 1 and 2 (Fig. 2). Notably, the computation time of SECREIT (11 s) was about 30 × shorter than those of Human 1 (326 s) and Human 2 (366 s).

**Table 3:** Confusion matrix of estrous stage classification by SECREIT and two human examiners using 100 test images without estrous stage cyclicity.

Ground truth	SECREIT			Human 1			Human 2		
	D	P	E	D	P	E	D	P	E
D	32	1	1	33	0	1	33	1	0
P	5	24	1	6	22	2	15	13	2
E	1	0	35	0	0	36	1	2	33

**Table 4:** Classification performance of the SECREIT and human examiners using 100 test images without estrous stage cyclicity.

	SECREIT			Human 1			Human 2		
	D (%)	P (%)	E (%)	D (%)	P (%)	E (%)	D (%)	P (%)	E (%)
Sensitivity	94.1	80.0	97.2	97.1	73.3	100.0	97.1	43.3	91.7
Specificity	90.9	98.6	96.9	90.9	100.0	95.3	75.8	95.7	96.9

**Figure 3:**
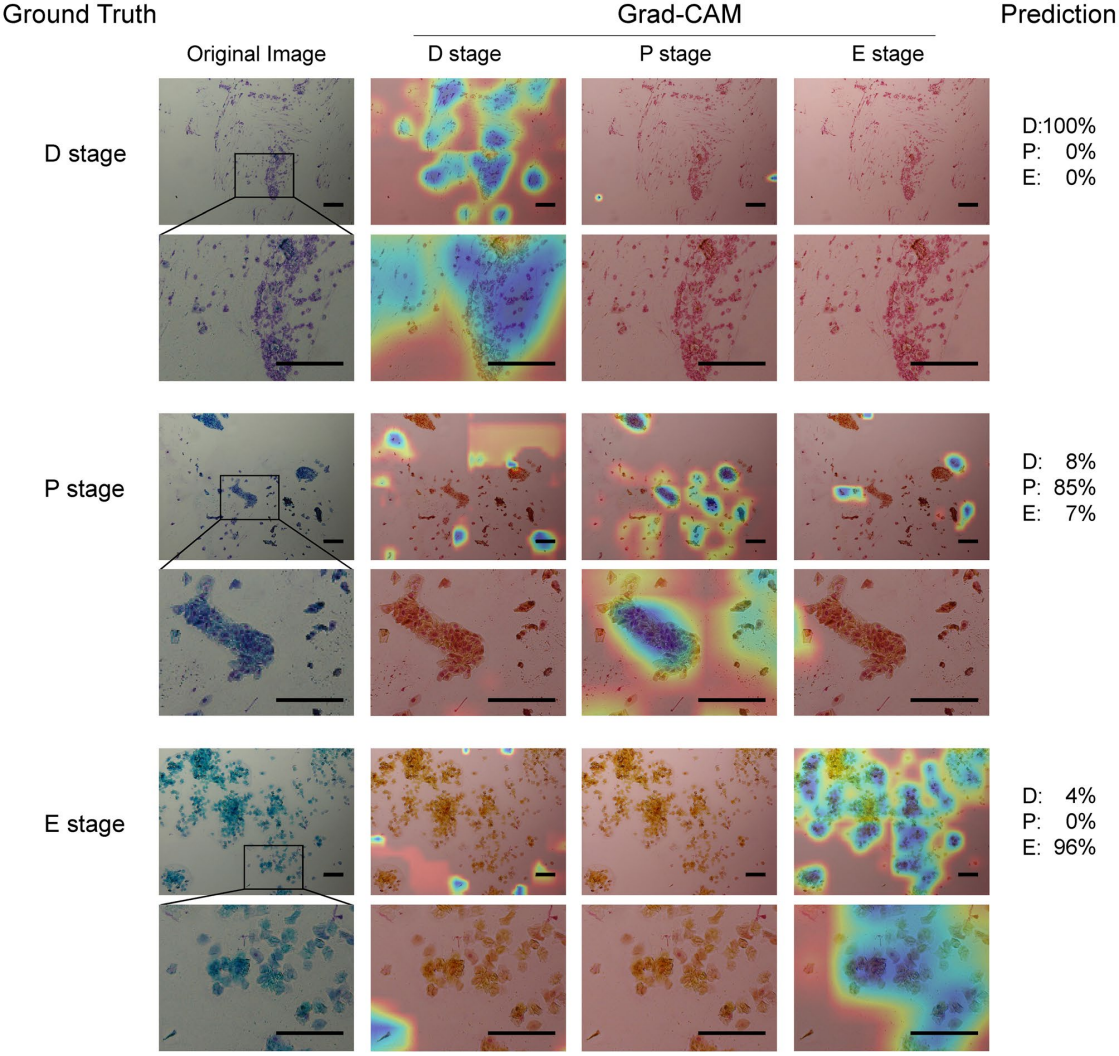
Features that contributed to correct classification by SECREIT. The heatmap images (right three columns) created by Grad-CAM are overlaid on the original microscopy image (leftmost column). The Grad-CAM (D stage), Grad-CAM (P stage), and Grad-CAM (E stage) columns represent the places that SECREIT estimates as features of the D, P, and E stages, respectively. SECREIT outputs the estimated probability of estrous stage (Prediction). The heatmap images revealed that SECREIT correctly classified these stages by the presence of the characteristic cell types, just as the human examiners did. Scale bars represent 100 μm.

The important parts in the tested images that contributed to SECREIT's prediction were visualized as heatmap images (Fig. 3), which revealed that SECREIT identified each cell type. Stage D was identified by the presence of leukocytes and nucleated cells, Stage P was identified by the presence of nucleated epithelial cells and cornified cells, and stage E was identified by the presence of nucleated epithelial cells.

## Discussion

In this study, we developed an automatic estrous cycle stage classifier with a deep learning algorithm, and the results of our analyses demonstrated that the model achieved high sensitivity, specificity, and AUCs. The test using 100 random images showed that the accuracy of SECREIT was comparable to that of experienced human examiners. Once trained, SECREIT can classify the images significantly faster than human examiners. As recommended for NIH-funded research^11^, the number of preclinical studies using female rodents will continue to increase, and for the interpretation of data obtained in animal and human females, it is very important to determine the estrous cycle. As we noted earlier, the determination of rodent estrous cycle stages by human examiners requires a long training period, takes a long time, and produces results that may not match among multiple examiners. The SECREIT can be used to meet the increasing demands for determining the estrous stages of female animal.

SECREIT showed the same classification and misclassification tendencies as those shown by the humans in this study. Although SECREIT showed a low sensitivity for the P stage compared to the other stages, its sensitivity for this stage was higher than that of the two humans (Table 4). SECREIT had a tendency to misclassify the P images as stage D, which was also consistent with the humans' misclassification (Table 3). Discrimination between stages D and P from a vaginal smear image is often difficult for human examiners because the types and proportions of cells in the latter phase of D are similar to those in the P stage^12^. Grad-CAM revealed that SECREIT may identify mucus, dust, or less-stained nucleated epithelial cells as leukocytes in this misclassification (Supplementary Fig. S2). Increasing the stage P images for training might reduce the rate of this misclassification.

We compared a VGG16-based model using transfer learning with CBR-LargeT, a light weight model, trained from a scratch. The experimental results showed the VGG16-based model outperformed the CBR-LargeT model, which is inconsistent with the observation that transfer learning doesn’t result in better performance in some medical image tasks due to the different characteristics of general images in ImageNet and the medical images^25^. One of a few problems of transfer learning for medical image pointed out is that “many medical imaging tasks start with a large image of a bodily region of interest and use variations in local textures to identify pathologies”. Comparatively homogeneous cytology images, rather than bodily images taken by X-ray, a computed tomography, or a funduscope, might be a reason why transfer learning had a positive effect in our study.

Depending on the researchers and the objectives of a study, the estrous cycle is divided into three or four stages^12^. In the present investigation, we adopted the three-stage classification because the metestrus stage is shorter (6–8 h) than the other stages (D: 48–72 h: P: ~ 14 h, and E: 12–48 h)^17^, and it was difficult to acquire enough images to train for a four-stage classification. We used images from a single laboratory and a single species herein, but there are differences among laboratories regarding the sample fixation, staining procedures, imaging, and scanners, and differences concerning cell features across species and strains^17^, all of which could adversely affect the accuracy of the computational analysis. Further evaluations of SECREIT are thus required. However, SECREIT achieved very high accuracy and showed the level of practical use in classifying the estrous cycle stage of mice based on smear images. The SECREIT can thus become a first step toward accelerating research that uses female mice.

## Materials and methods

### Animals

A total of 664 female mice and 3,319 microscopic images were amassed (Supplementary Table S1). Female C57BL/6J mice (5–14 weeks of age) were purchased from Japan SLC (Shizuoka, Japan). The mice were provided food and water ad libitum and maintained on a 12-h light/dark cycle throughout the study. All animal-use procedures were in accord with the Guidelines for Animal Experimentation of Showa Pharmaceutical University. According to the guidelines for Animal Experimentation of Chiba University, the need for ethical approval was waived.

### Vaginal cytology methods

A vaginal swab was collected from each mouse with a cotton tipped swab (Asone, Osaka, Japan) wetted with 0.9% saline and inserted into the vagina. The swab was gently turned and rolled against the vaginal wall and then removed. The cells on the swab were transferred to a dry slide glass. The slide was dried for ≥ 1 day and then stained with 4% Giemsa stain solution for 25 min at room temperature. The slides were rinsed with water. The images of cells were captured at 10 × objective lens under bright field illumination by a light microscope (BX50, Olympus, Tokyo) connected with a digital camera (Digital Sight DS-L3, Nikon Instech, Tokyo).

The vaginal swabs were collected from mice that were used in other unpublished behavioral studies in which the mice were injected with a drug or underwent an ovariectomy and/or contextual or cued fear conditioning. We confirmed that the injected drugs and behavioral tests did not influence the estrous cycles of the mice. The collection of vaginal swabs was conducted between 08:00 and 16:00 over 1–5 consecutive days. Regardless of when the samples were collected, it was done at approximately the same time of the day over the course of the collection period in each mouse to reduce variability.

The estrous cycle stage was manually determined by two experienced examiners (S.M. and S.T.) based on the percentages of leukocytes, cornified epithelial cells, and nucleated epithelial cells and the cyclicity as described^14,15,17^ (Fig. 1a–c). One of the examiners was 35 years old man and had judged 6,512 vaginal smear images over a 7-year period (Human 1), and the other was 28 years old man who had judged 3,233 images over a 3-year period (Human 2).

### Ovariectomy

Of the total of 664 mice, 323 underwent a bilateral ovariectomy or a sham surgery in the present study. The mice were anesthetized with a mixture of 0.18 mg/kg medetomidine hydrochloride (Wako, Osaka, Japan), 2.4 mg/kg midazolam (Wako), and 3 mg/kg butorphanol tartrate (Meiji Seika Pharma, Tokyo). The three-mix anesthetic was injected subcutaneously (6 μl/g). At ≥ 1 week after the surgery, we performed the vaginal cytology experiment, and we confirmed that the cyclicity had stopped in the ovariectomized mice and remained at a stage resembling diestrus (Fig. 1d).

### Datasets

We used 2,096 microscopy images from the vaginal smears (D: n = 1,476, E: n = 449, P: n = 171) for the training datasets, 487 images (D: n = 314, E: n = 137, P: n = 36) for the validation datasets, and 736 images (D: n = 486, E: n = 183, P: n = 67) for the test datasets. These training, validation, and test datasets were taken from different experiments. Fifty-six images (training: n = 38 images, validation: n = 11, test: n = 7) judged as an intermediate class between [D and P], [P and E], or [E and D] stage by the above-cited examiners were used for training as both pairs of classes but were excluded from the validation and test datasets. In addition, the stages of 27 images (training: n = 9 images, validation: n = 7, test: n = 11) could not be determined, and these images were excluded from all three datasets. The original images were 960 × 1,280 pixels, and we divided them into four 480 × 640 pixel images and resized these to 240 × 320 pixels for their input into the deep learning model.

### SECREIT’s architecture and model training

**Figure 4:**
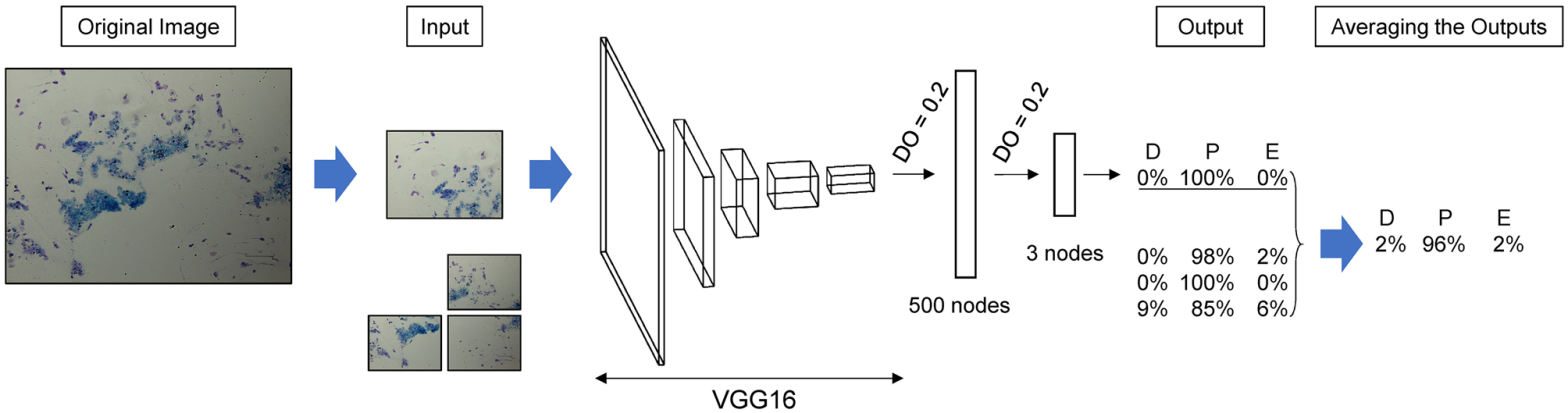
Overview of SECREIT model. Each microscopic image was divided into four images. The convolutional neural network consisted of VGG16 and two fully connected layers. The averaged probability scores from four images were used to evaluate the model. *DO* dropout rate.

The deep learning model was written in Python (ver. 3.6.7) and Keras (ver. 2.2.4), a Python-based open-source deep learning framework, and with TensorFlow (ver. 1.14.0, Google) as its backend. Our model consisted of 13 convolutional layers based on VGG16 and two fully connected layers (dropout = 20%, each)^24,26^, which consisted of 500 nodes and three nodes, respectively (Fig. 4, Supplementary Table S2).

The input of the model was the 240 × 320-pixel images, each of which was one of the four images divided from an original image. Each divided image's probability of estrous stage was estimated and averaged as the probability score of the original image (Fig. 4). The averaged probability scores were used in the validation and the test.

During the training, the images were augmented. Each image was rotated randomly between 0° and 180°, flipped with a probability of 0.5, scaled horizontally and vertically from 0.9 times to 1.1 times, with a change in shear intensity from 0.9 times to 1.1 times, a change in brightness from 0.5 times to 1.0 times, and a random change in the RGB intensity in the range of 20. The input means were set to 0 over the dataset, feature-wise, and ZCA whitening was applied. The training images of each stage were sampled with equal probability to reduce the effect of class imbalance.

First, the network parameters were initialized to the best parameter set that was achieved in ImageNet competition, and only the last two layers of the pre-trained model were trained for 50 epochs. The model with the best validation accuracy was recorded. Then, all the layers of the best model were retrained for 100 epochs. Finally, we selected the best parameter set for test, which showed ≥ 65% sensitivity in any estrous stage (D, P, and E) and the highest average accuracy in validation dataset. A categorical hinge was used as the loss function and Nadam optimization^27^ with the learning rate of 2 × 10^–5^. It takes 1.4 h for all training.

### CBR-LargeT architecture and training

CBR-LargeT consisted of 5 convolutional layers and a fully connected layer (Supplementary Table S3), and the model is trained for 100 epochs from a scratch. Data augmentation and sampling protocol is the same with those of VGG16-based model, as described above. A categorical hinge was used as the loss function and Adam optimization^28^ with the learning rate of 1 × 10^–3^. The best parameter set, which showed ≥ 65% sensitivity in any estrous stage (D, P, and E) and the highest average accuracy in validation dataset, was selected for test.

### Statistical analyses

We evaluated the performance of the SECREIT by using the test dataset. Sensitivity and specificity were calculated for each estrous stage, and we computed the ROC curve and corresponding area under ROC curve (AUC) for each estrous stage by using the open source Python library scikit-learn. We also compared the performance and consumption times of SECREIT and the two human examiners by using 100 images without estrous stage cyclicity. One hundred images were randomly sampled from the test dataset: 34 images of D stage, 30 images of P stage, and 36 images of E stage. The overall accuracy of the human examiners and the SECREIT was calculated as the correct answer rate in these 100 test images. The SECREIT consumption computation time was measured using one Quadro GV100 GPU and Dual Intel Xeon Platinum 8176 CPU 2.10 GHz.

### Visual explanation of SECREIT's decisions

To understand how the SECREIT worked, we visualized the important places that contributed to SECREIT's predictions by obtaining a heatmap with Gradient-weighted Class Activation Mapping (Grad-CAM)^29^. The gradients of each estrous stage's probability score with respect to each place's output of the last convolutional were calculated and smoothed for visualization. We evaluated the heatmap in both the successful and failed classifications, and we compared the heatmap with the important places cited by the human judges.

## Supplementary information

Supplementary Information

## Data Availability

The datasets analyzed during this study are available at https://opac.ll.chiba-u.jp/da/curator/108041/. The SECREIT is available at https://github.com/SanoKyohei/Secreit.
