# Supplementary material for: Deep learning-based classification of the mouse estrous cycle stages
Source: Sci Rep. 2020 Jul 16;10:11714. doi: 10.1038/s41598-020-68611-0 (PMC7366650; doi:10.1038/s41598-020-68611-0)
Supplement: Supplementary file 1 — Supplementary Information [file 41598_2020_68611_MOESM1_ESM.pdf]

Deep learning-based classification of the mouse estrous cycle stages

Supplementary Information

Kyohei Sano, Shingo Matsuda, Suguru Tohyama,
Daisuke Komura, Eiji Shimizu, Chihiro Sutoh

Supplementary Table S1. Characteristic of the datasets

Profile of animals	No. of animals	Image			
		D	P	E	Total
Non-treated	53	175	24	67	266
Sham/Ovariectomized	323	1310	83	254	1647
Vehicle/drug-administered	288	791	167	448	1406
Total	664	2276	274	769	3319

Supplementary Table S2. Architecture of the SECREIT model

	Layer #	Layer (type)	Output shape
Input		input_1 (InputLayer)	(None, 240, 320, 3)
VGG16	1	block1_conv1 (Conv2D)	(None, 240, 320, 64)
	2	block1_conv2 (Conv2D)	(None, 240, 320, 64)
		block1_pool (MaxPooling2D)	(None, 120, 160, 64)
	3	block2_conv1 (Conv2D)	(None, 120, 160, 128)
	4	block2_conv2 (Conv2D)	(None, 120, 160, 128)
		block2_pool (MaxPooling2D)	(None, 60, 80, 128)
	5	block3_conv1 (Conv2D)	(None, 60, 80, 256)
	6	block3_conv2 (Conv2D)	(None, 60, 80, 256)
	7	block3_conv3 (Conv2D)	(None, 60, 80, 256)
		block3_pool (MaxPooling2D)	(None, 30, 40, 256)
	8	block4_conv1 (Conv2D)	(None, 30, 40, 512)
	9	block4_conv2 (Conv2D)	(None, 30, 40, 512)
	10	block4_conv3 (Conv2D)	(None, 30, 40, 512)
		block4_pool (MaxPooling2D)	(None, 15, 20, 512)
	11	block5_conv1 (Conv2D)	(None, 15, 20, 512)
	12	block5_conv2 (Conv2D)	(None, 15, 20, 512)
	13	block5_conv3 (Conv2D)	(None, 15, 20, 512)
		block5_pool (MaxPooling2D)	(None, 7, 10, 512)
FCL		flatten_1 (Flatten)	(None, 35840)
		dropout_1 (Dropout)	(None, 35840)
	14	dense_1 (Dense)	(None, 500)
		dropout_2 (Dropout)	(None, 500)
Output	15	dense_2 (Dense)	(None, 3)

FCL: Fully connected layers.

Supplementary Table S3. Architecture of the CBR-LargeT model

	Layer #	Layer (type)	Output shape
Input		input_1 (InputLayer)	(None, 240, 320, 3)
	1	block1_conv (Conv2D)	(None, 234, 314, 32)
		block1_norm (Batch normalization)	(None, 234, 314, 32)
		block1_relu (Relu)	(None, 234, 314, 32)
		block1_pool (MaxPooling2D)	(None, 116, 156, 32)
	2	block2_conv (Conv2D)	(None, 110, 150, 64)
		block2_norm (Batch normalization)	(None, 110, 150, 64)
		block2_relu (Relu)	(None, 110, 150, 64)
		block2_pool (MaxPooling2D)	(None, 54, 74, 64)
	3	block3_conv (Conv2D)	(None, 48, 68, 128)
		block3_norm (Batch normalization)	(None, 48, 68, 128)
		block3_relu (Relu)	(None, 48, 68, 128)
		block3_pool (MaxPooling2D)	(None, 23, 33, 128)
	4	block4_conv (Conv2D)	(None, 17, 27, 256)
		block4_norm (Batch normalization)	(None, 17, 27, 256)
		block4_relu (Relu)	(None, 17, 27, 256)
		block4_pool (MaxPooling2D)	(None, 8, 13, 256)
	5	block5_conv (Conv2D)	(None, 2, 7, 512)
		block5_norm (Batch normalization)	(None, 2, 7, 512)
		block5_relu (Relu)	(None, 2, 7, 512)
		block5_pool<br>(Global Average Pooling2D)	(None, 512)
Output, FCN	6	dense (Dense)	(None, 3)

FCL: Fully connected layers.

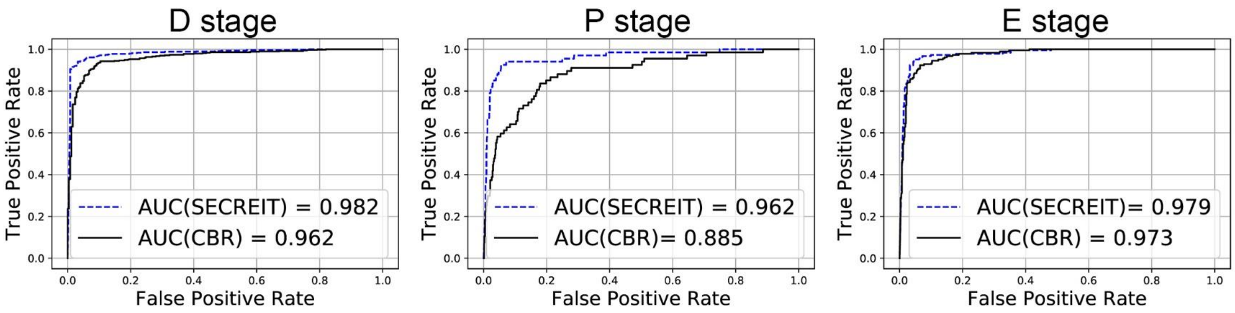

Supplementary Figure S1. Comparison of the accuracy indices for the VGG16-based model and the CBR-LargeT model.

The ROC curves for the VGG16-based model (SECREIT) and the CBR-LargeT model are illustrated.

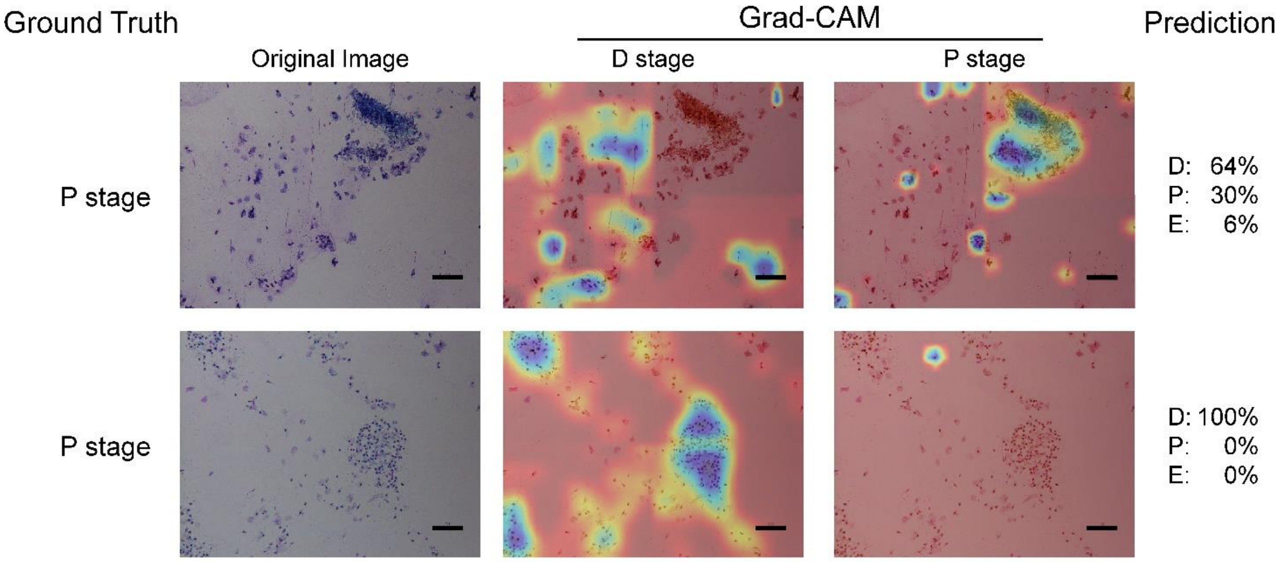

Supplementary Figure S2. Features that lead SECREIT to misclassification.

The heatmap images (*right two columns*) created by Grad-CAM are overlaid on the original microscopy image (*leftmost column*). The Grad-CAM (D stage) and Grad-CAM (P stage) columns represent the places that SECREIT estimates as features of the D and P stages, respectively. SECREIT outputs the estimated probability of the estrous stage (Prediction). The heatmap images revealed that SECREIT identified mucus, dust, and less-stained nucleated epithelial cells as leukocytes and misclassified P images as stage D. Scale bars represent 100 micrometers.
